# Differential Growth Responses of Marine Phytoplankton to Herbicide Glyphosate

**DOI:** 10.1371/journal.pone.0151633

**Published:** 2016-03-17

**Authors:** Cong Wang, Xin Lin, Ling Li, Senjie Lin

**Affiliations:** 1 State Key Laboratory of Marine Environmental Science and College of Ocean and Marine Biodiversity and Global Change Research Center, Xiamen University, Xiamen, Fujian, China; 2 Department of Marine Sciences, University of Connecticut, Groton, Connecticut, United States of America; National Taiwan Ocean University, TAIWAN

## Abstract

Glyphosate is a globally popular herbicide to kill weeds and its wide applications may lead to accumulation in coastal oceans as a source of phosphorus (P) nutrient or growth inhibitor of phytoplankton. We studied the physiological effects of glyphosate on fourteen species representing five major coastal phytoplankton phyla (haptophyta, bacillariophyta, dinoflagellata, raphidophyta, and chlorophyta). Based on growth responses to different concentrations of glyphosate under contrasting dissolved inorganic phosphorus (DIP) conditions, we found that phytoplankton species could be classified into five groups. Group I (*Emiliania huxleyi*, *Skeletonema costatum*, *Phaeodactylum tricornutum*) could utilize glyphosate as sole P-source to support growth in axenic culture, but in the presence of DIP, they were inhibited by both 36-μM and 360-μM glyphosate. Group II (*Karenia mikimotoi*, *Prorocentrum minimum*, *Dunaliella tertiolecta*, *Symbiodinium* sp., *Heterosigma akashiwo* and *Alexandrium catenella*) could not utilize glyphosate as sole P-source to support growth, and in the presence of DIP growth was not affected by 36-μM but inhibited by 360-μM glyphosate. Glyphosate consistently enhanced growth of Group III (*Isochrysis galbana*) and inhibited Group IV (*Thalassiosira weissflogii*, *Thalassiosira pseudonana* and *Chattonella marina*) regardless of DIP condition. Group V (*Amphidinium carterae*) exhibited no measurable response to glyphosate regardless of DIP condition. This grouping is not congruent with the phylogenetic relationships of the phytoplankton species suggesting functional differentiation driven by environmental pressure. We conclude that glyphosate could be used as P-source by some species while is toxic to some other species and yet has no effects on others. The observed differential effects suggest that the continued use of glyphosate and increasing concentration of this herbicide in the coastal waters will likely exert significant impact on coastal marine phytoplankton community structure.

## Introduction

Organophosphonate herbicide glyphosate [N-(phosphonomethyl) glycine] [1] is a chemically-synthesized compound, also a type of dissolved organic phosphorus (DOP) that contains a stable C-P bond. It has become a global herbicide in agriculture because of its outstanding performances. After entering plants, glyphosate inhibits the activity of 5-enolpyruvylshikimate-3-phosphate (EPSP) synthase [2], a key enzyme for the synthesis of aromatic amino acids, and causes cell death [3]. In addition to this main mode of action, glyphosate is also known to damage a number of cellular structures and other biochemical processes, such as disruption of chloroplasts, membranes and cell walls, reduction in chlorophyll content and changes in nucleic acid synthesis, photosynthesis, and respiration [4–6]. These characteristics render glyphosate to be one of the most popular agricultural herbicides [7]. Because animals do not have these targets of glyphosate action, this herbicide is widely viewed as environmentally benign [8,9].

The usage of glyphosate is notably high worldwide. It has been reported that typical forestry and agricultural application rates of glyphosate-based herbicides range from 0.9 to 4.27 kg acid equivalents (a.e.)/ha, and in the United States annual application is up to 6.73 kg a.e./ha for crop uses and 8.92 kg a.e./ha for noncrop uses [10]. Direct overspraying a 15-cm deep wetland with no intercepting vegetation at these application rates has been estimated to result in aqueous concentrations between 2.89 (about 17.34 μM, at the maximum label application rate) and 5.95 mg/L (about 35.7 μM, one time application of the maximum annual application rate) [11] either by accidental or wind driven drift of the herbicide spray, or by surface runoff of suspended particulate matter [12–14]. As an unnatural chemical herbicide, its potential effects on the aquatic ecosystem should be given attention [15–17]. Although many factors such as the pH, water alkalinity and trophic state may cause variability in glyphosate concentrations, the wide applications of glyphosate and its relatively long half-life (7 to 315 days, most commonly 45–60 days) will lead to its constant presence in coastal waters [18]. Several previous studies have characterized the effects of individual glyphosate-based herbicide formulations on a wide variety of aquatic organisms, including microorganisms [19,20], invertebrates [21,22], amphibians [11,23], and fish [24–26], which indicated diverse physiological and behavioral effects depending on the dose and formulation. However, relatively few investigations have been published on the effects of glyphosate on marine phytoplankton [3,27,28]. It is important to assess the potential impact on phytoplankton, considering the vital ecological roles of these photosynthetic organisms in the marine ecosystem. Evidence is available that glyphosate has direct toxic effects on populations of phytoplankton [29]. Furthermore, the adverse effects on the primary producers can be cascaded to higher trophic levels and hence the function of the entire ecosystem may be impacted [30].

Despite the toxic effects on weeds, glyphosate can be utilized by microbial communities as an alternative source of C, N or P [31–33], which is essential to all living organisms. Many studies have indicated that some bacteria, actinomycetes, fungi and unidentified microbes can degrade glyphosate [34,35]. *Sinorhizobium meliloti* of the family Rhizobiaceae, for instance, has been shown to be able to utilize glyphosate naturally as sole P-source [36]. Numerous studies over the past two decades have provided evidence that P is the ultimate limiting nutrient of phytoplankton growth in oceanic as well as some coastal waters [37–40] and even terrestrial ecosystems [41,42]. This is because phosphate minerals are sparingly soluble ([PO_4_^-3^] = 1 mM at pH 7, 25°C), and geochemical cycling of phosphate is slow, making the concentration of orthophosphate, the form of P that is immediately available to organisms, very low. Therefore, DOP often serves as an alternative P-source to support the growth of marine phytoplankton [43,44]. Many studies have been conducted to understand phytoplankton utilization of phosphorus esters, which contribute 75% of high-molecular-weight DOP pool in marine systems [45]. However, for the remaining 25% DOP [46–48], phosphonates, to which glyphosate belongs, we know little about its potential to be utilized as a P-source by phytoplankton.

In this study, we investigated the effects of glyphosate on phytoplankton growth under different P conditions, to assess whether glyphosate can support the growth of phytoplankton or inhibit their growth as an herbicide. Our results showed that different phytoplankters responded differently to glyphosate.

## Materials and Methods

### Algal cultures

Fourteen phytoplankton species obtained from Collection Center of Marine Algae (Xiamen University, China) and belonging to five different phyla (i.e. haptophyta, bacillariophyta, dinoflagellata, raphidophyta, and chlorophyta) were selected for the experiments (Table 1). *Isochrysis galbana* and *Emiliania huxleyi* from the haptophyta group provide important nutritional values as commonly used pray [49] and produce calcites as well as dimethylsulfoniopropionate (DMSP), respectively [50]. The diatoms *Skeletonema costatum*, *Phaeodactylum tricornutum*, *Thalassiosira weissflogii*, and *Thalassiosira pseudonana* are all common coastal phytoplankters [51], among which, *P*. *tricornutum* and *T*. *pseudonana*, are two best studied model species [52,53] with genomes fully sequenced [54,55]. The dinoflagellates *Alexandrium catenella*, *Prorocentrum minimum*, *Kerania mikimotoi*, and *Amphidinium carterae* as well as the raphidophytes *Heterosigma akashiwo* and *Chattonella marina* cause harmful algal blooms under certain nutrient (e.g. increasing phosphorus availability) and climate conditions (increasing temperature) [56], and all of these can produce toxins. Many species of the dinoflagellate genus *Symbiodinium* are essential endosymbionts of the reef-building corals [57]. *Dunaliella tertiolecta* was included because it is often used as a model marine chlorophyte, and its photosynthetic apparatus is similar to those of higher plants [58,59].

**Table 1:** Fourteen algal species examined in our study and summary (and grouping) of their differential responses to glyphosate.

Phylum	Species	Sole P-source*	+DIP+lower glyphosate*	+DIP+higher glyphosate*	Grouping
Haptophyta	*Isochrysis galbana*	+	–	promote	III
	*Emiliania huxleyi*	+	inhibit	inhibit	I
Bacillariophyta (Diatoms)	*Skeletonema costatum*	+	inhibit	inhibit	I
	*Phaeodactylum tricornutum*	+	inhibit	inhibit	I
	*Thalassiosira weissflogii*	inhibit	inhibit	inhibit	IV
	*Thalassiosira pseudonana*	inhibit	inhibit	inhibit	IV
Dinoflagellata	*Alexandrium catenella*	–	–	inhibit	II
	*Prorocentrum minimum*	–	–	inhibit	II
	*Karenia mikimotoi*	–	–	inhibit	II
	*Symbiodinium* sp.	–	–	inhibit	II
	*Amphidinium carterae*	–	–	–	V
Raphidophyta	*Heterosigma akashiwo*	–	–	inhibit	II
	*Chattonella marina*	inhibit	inhibit	inhibit	IV
Chlorophyta	*Dunaliella tertiolecta*	–	–	inhibit	II

*In these columns, “+” represents that glyphosate could be used as sole P-source (*p*<0.05, RM ANOVA); “–” represents no effect; “inhibit” or “promote” represents glyphosate could inhibit or promote growth (*p*<0.05, RM ANOVA).

Stock cultures of these species were grown in f/2 or L1 medium (without silicate) with 0.22 μm-filtered and autoclaved seawater (30 salinity) at 20°C under 12h: 12h light: dark photocycle with a photon flux of 120 μmol·m^-2^·s^-1^. Cell concentrations were measured microscopically using a Sedgewick-Rafter counting chamber (Phycotech, St. Joseph, MI, USA) following previous reports[60–62], to monitor the growth of these cultures. Experiments with glyphosate began when the stock cultures entered the exponential growth stage.

### Experimental design

Stock solution of glyphosate (99.9%, non-derived compound; SIGMA-ALDRICH) was dissolved in Milli-Q water to a concentration of 36 mM, and then sterile-filtered through a 0.22-μm membrane and stored at 4°C. Seawater used in this study was open ocean water collected from the South China Sea where background DIP concentration was 0.73 μM, which was filtered through a 0.45-μM membrane and autoclaved.

Three laboratory experiments were conducted to investigate two different issues. Experiment I and Experiment III were designed to evaluate whether the examined phytoplankton species are able to degrade glyphosate to phosphate (P-source), while the primary objective of Experiment II was to assess the toxic effects of glyphosate at 36 μM (6 mg·L^-1^) and 360 μM (60 mg·L^-1^) concentrations (most aquatic organisms had high glyphosate tolerance [63]). Experiment I composed the +DIP control (f/2 or L1 medium, with 36 μM DIP), glyphosate-amended (provided at 36 μM as sole P-source in the f/2 or L1 minus DIP medium), and the–DIP treatment (without any added P-source). Experiment II shared the +DIP control group with Experiment I, but in addition had two other treatments, respectively added with 36 μM and 360 μM glyphosate in the normal f/2 or L1 medium (i.e. containing 36 μM DIP) (Table 2). Each treatment was conducted in triplicate, and all experiments were run in 400-mL contained in 600-mL flasks. In the first round of experiment, cultures were not axenic. In the second round of experiment, we set up an Experiment III (Table 2) to re-examine four of the species used in Experiment I, including the haptophyte *I*. *galbana* and *E*. *huxleyi*, and the diatoms *S*. *costatum* and *P*. *tricornutum*, in which we not only acquired axenic cultures with 50g·L^-1^ antibiotics (ampicillin, streptomycin, and kanamycin) treatment, but also set up a serial concentrations of glyphosate (0.36 μM, 3.6 μM, and 36 μM) to cover the range previously found in the field, 0.60 to 4.2 μM [64]. We checked the cultures by DAPI staining and epifluorescence microscopic examination at the beginning and end of each treatment, and verified that no bacteria were seen throughout the experimental period. The general glyphosate concentration regime was designed also with consideration of the phosphorus concentration in the normal f/2 or L1 medium (36 μM), and was made comparable to the concentrations assayed by other authors [15,20,35,65–74].

**Table 2:** P additions for each treatment in Experiments I-III.

P-source	Control	Experiment I		Experiment II		Experiment III			
DIP (μM)	36	0	0	36	36	36	0	0	0
Glyphosate (μM)	0	0	36	36	360	0	0.36	3.6	36

### Growth monitoring and DIP measurement

In the course of the experiments, samples were collected periodically until stationary phase was reached for obtaining cell concentrations and DIP concentrations in the cultures. Cell count was conducted as described above; at the same time, a 25-mL sample was filtered through 0.45-μm mixed cellulose ester membrane, and soluble reactive phosphorus in the filtrate was measured following the Phosphorus Molybdenum Blue Method [75].

### Statistical analysis

For all parameters, differences among treatments were assayed using repeated-measures analysis of variance (RM ANOVA) [76], with five treatments. RM ANOVA analyses were followed by all pairwise multiple comparisons (post hoc testing), using the Holm-Sidak method.

## Results

### Glyphosate as sole P-source

**Fig 1:**
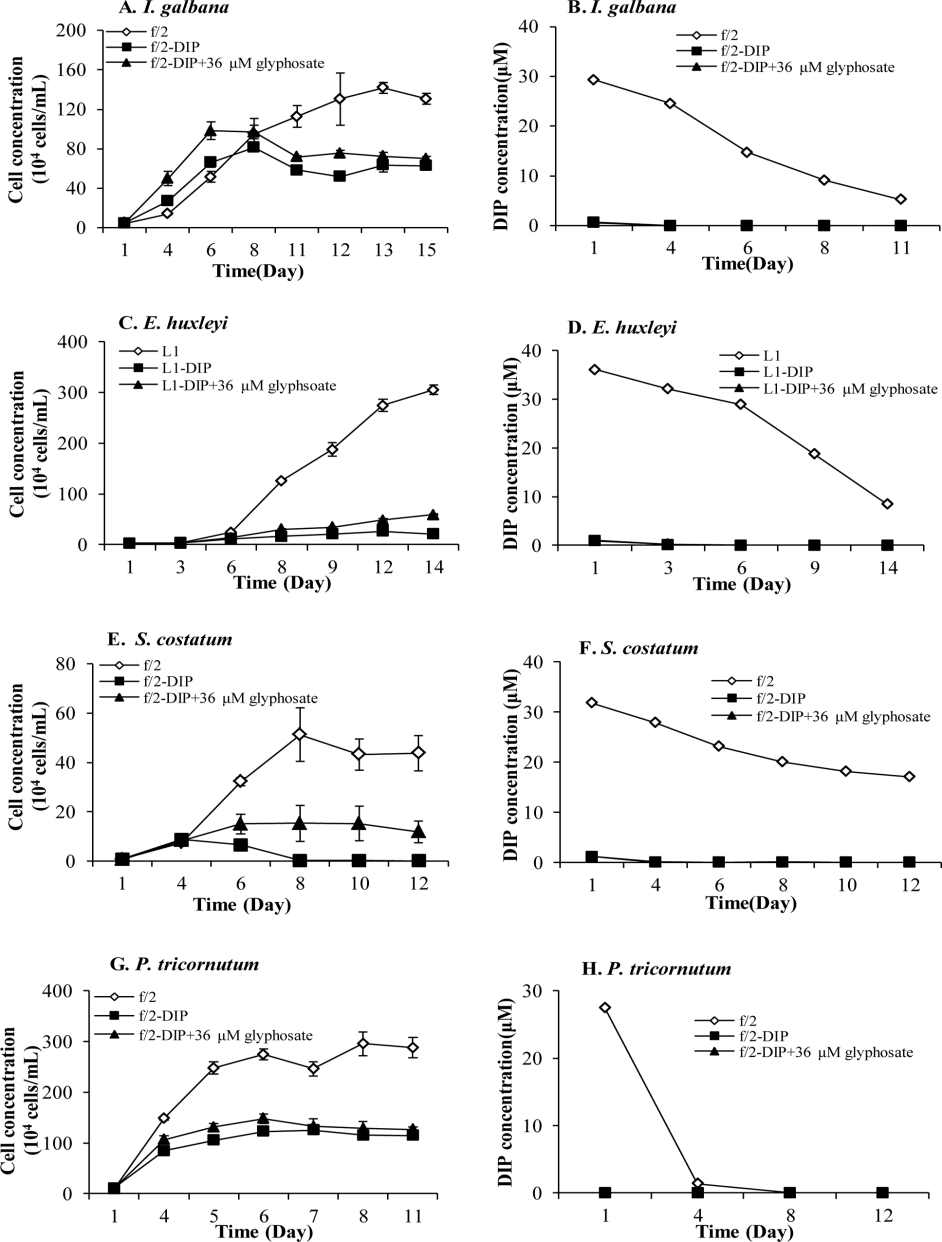
Growth responses of phytoplankton species to glyphosate in Group I and Group III. (A) (C) (E) (G) Growth curves of Group I (*E*. *huxleyi*, *S*. *costatum*, *P*. *tricornutum*) and Group III (*I*. *galbana*), whose growth were supported in–DIP+36μM glyphosate cultures compared to–DIP cultures. (B) (D) (F) (H) No DIP release during the whole growth period in–DIP+36μM glyphosate cultures. Error bar represents standard deviation.

We examined fourteen phytoplankton species in Experiment I to explore whether they could utilize glyphosate as sole P-source to support growth, by measuring cell densities and DIP concentrations in the growth media during the approximately two-week experimental period. Four species, including *I*. *galbana*, *E*. *huxleyi*, *S*. *costatum* and *P*. *tricornutum* grew better in the cultures with 36-μM glyphosate as P-source than in the–DIP treatment (Fig 1A, 1C, 1E and 1G). However, the differences in the final cell yields were small between the two treatments. Both growth rates and biomass yields in the 36-μM glyphosate treatment were much lower than that in the +DIP control. Moreover, DIP concentrations (Fig 1B, 1D, 1F and 1H) in both treatments were nearly zero throughout the experimental period, indicating that the differences between them were due to the effects of glyphosate. Some differences were noted among the species in response to DIP limitation and the presence of glyphosate. In *I*. *galbana* (Fig 1A), the cultures in the two treatments without DIP initially grew slightly faster than the +DIP control group, suggesting that *I*. *galbana* is better adapted to phosphate limitation (than other species), likely a result of high-affinity phosphate uptake. Although *I*. *galbana* in the glyphosate treatment group, like that in the–DIP treatment group, entered the stationary growth phase much earlier than the +DIP control group, it maintained substantial growth and cell yield (~1/2 of yield in the +DIP control). In contrast, *S*. *costatum* cultures in all the three different P conditions initially grew similarly, but subsequently showed increasing differences among the three conditions, with the–DIP group almost dying out, and the 36-μM glyphosate treatment group grew to a substantial cell yield, which was however <1/2 that in the +DIP treatment group (Fig 1E). This suggests that *S*. *costatum* is less adapted to phosphorus limitation than *I*. *galbana*. In the cases of *E*. *huxleyi* and *P*. *tricornutum*, more moderate growth occurred in the–DIP treatment group, with small growth enhancement by the glyphosate treatment (Fig 1C and 1G).

**Fig 2:**
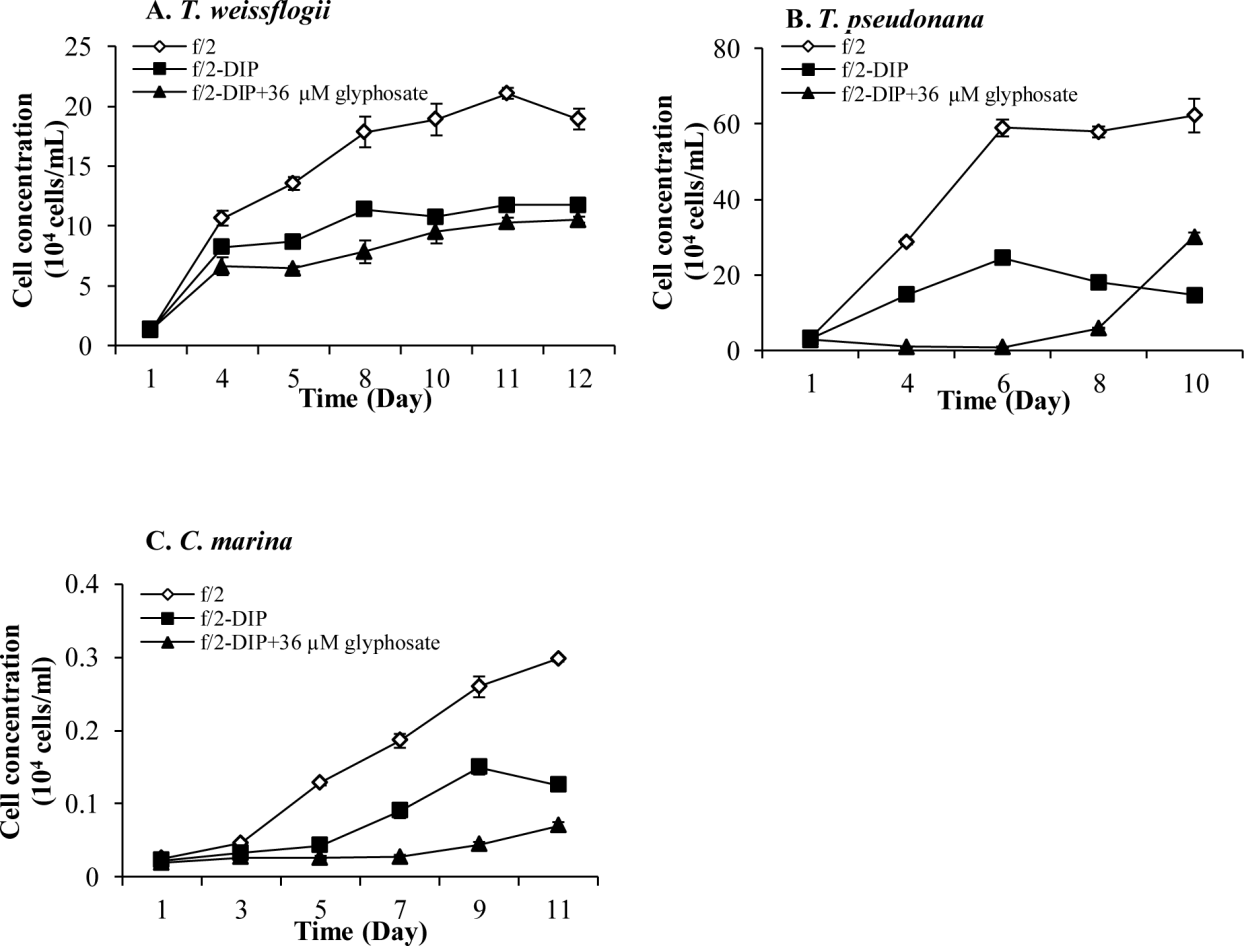
Growth responses of phytoplankton species to glyphosate in Group IV. (A) (B) (C) Growth curves of Group IV (*T*. *weissflogii*, *T*. *pseudonana*, *C*. *marina*), whose growth were inhibited significantly in–DIP+36μM glyphosate cultures compared to–DIP cultures. Error bar represents standard deviation.

Fig 2 displays three species (*T*. *weissflogii*, *T*. *pseudonana* and *C*. *marina*) whose growth in the 36-μM glyphosate treatment group was repressed even in comparison to the–DIP treatment group. For these three species, glyphosate likely acted as a toxic compound rather than a P-source. The only exception to this general trend was that for *T*. *pseudonana* (Fig 2B), after being inhibited by glyphosate in the first 8 days, growth increased significantly on day 10. Considering that the cultures were not axenic, the late-stage growth might be because glyphosate induced bacteria in the culture to degrade glyphosate and release DIP.

**Fig 3:**
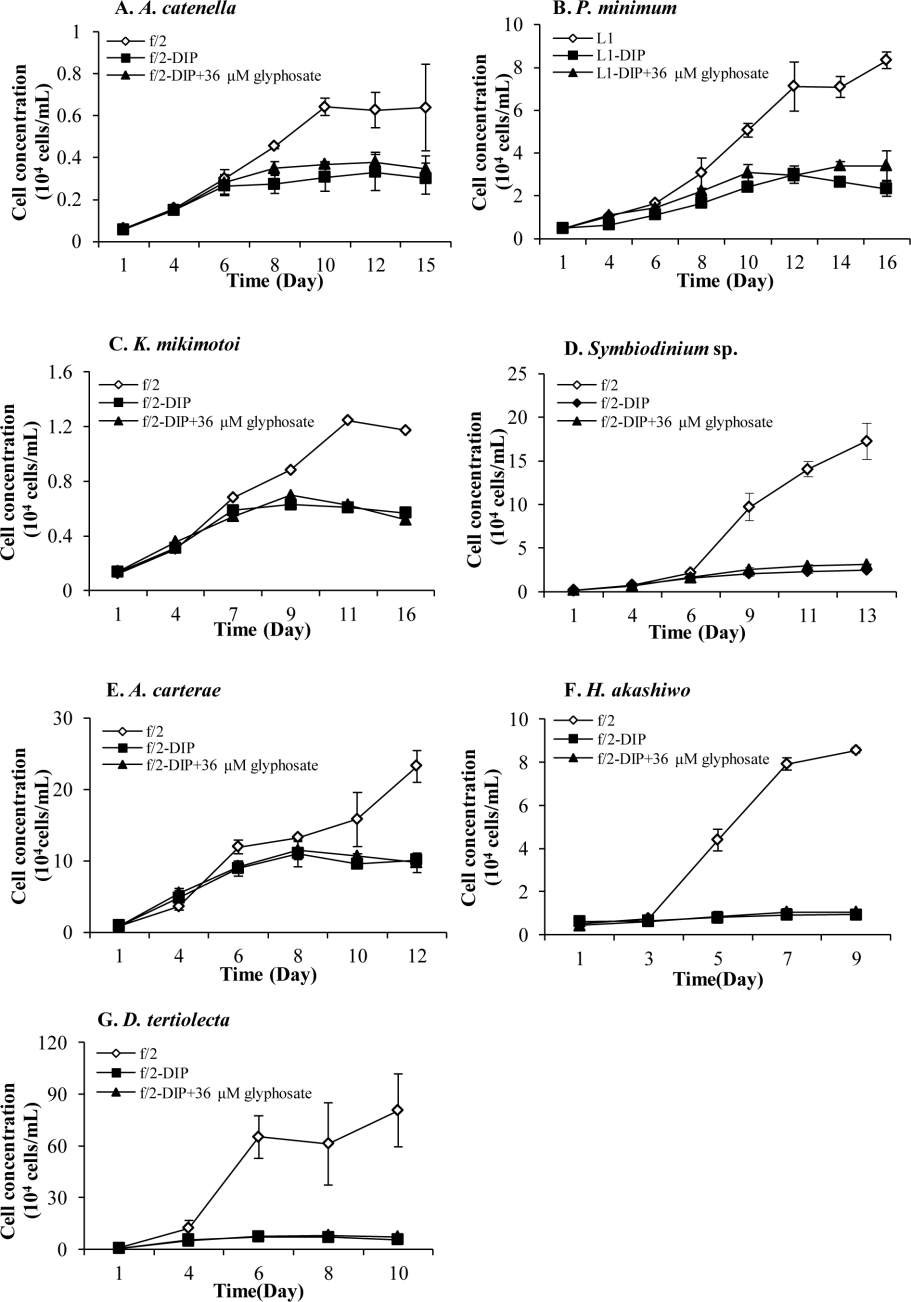
Growth responses of phytoplankton species to glyphosate in Group II and Group V. (A) (B) (C) (D) (E) (F) (G) Growth curves of Group II (A. catenella, P. minimum, *K*. *mikimotoi*, *Symbiodinium* sp., *H*. *akashiwo*, *D*. *tertiolecta*) and Group V (*A*. *carterae*), whose growth were not affected in the–DIP+36μM glyphosate cultures compared to the–DIP cultures. Error bar represents standard deviation.

The other seven species, *A*. *catenella*, *P*. *minimum*, *K*. *mikimotoi*, *Symbiodinium* sp., *A*. *carterae*, *H*. *akashiwo*, and *D*. *tertiolecta*, did not seem to use the added glyphosate (Fig 3). There were no significant differences in cell yield between the glyphosate treatment group and the–DIP group, both of which showed marked growth depression compared to the +DIP control group.

**Fig 4:**
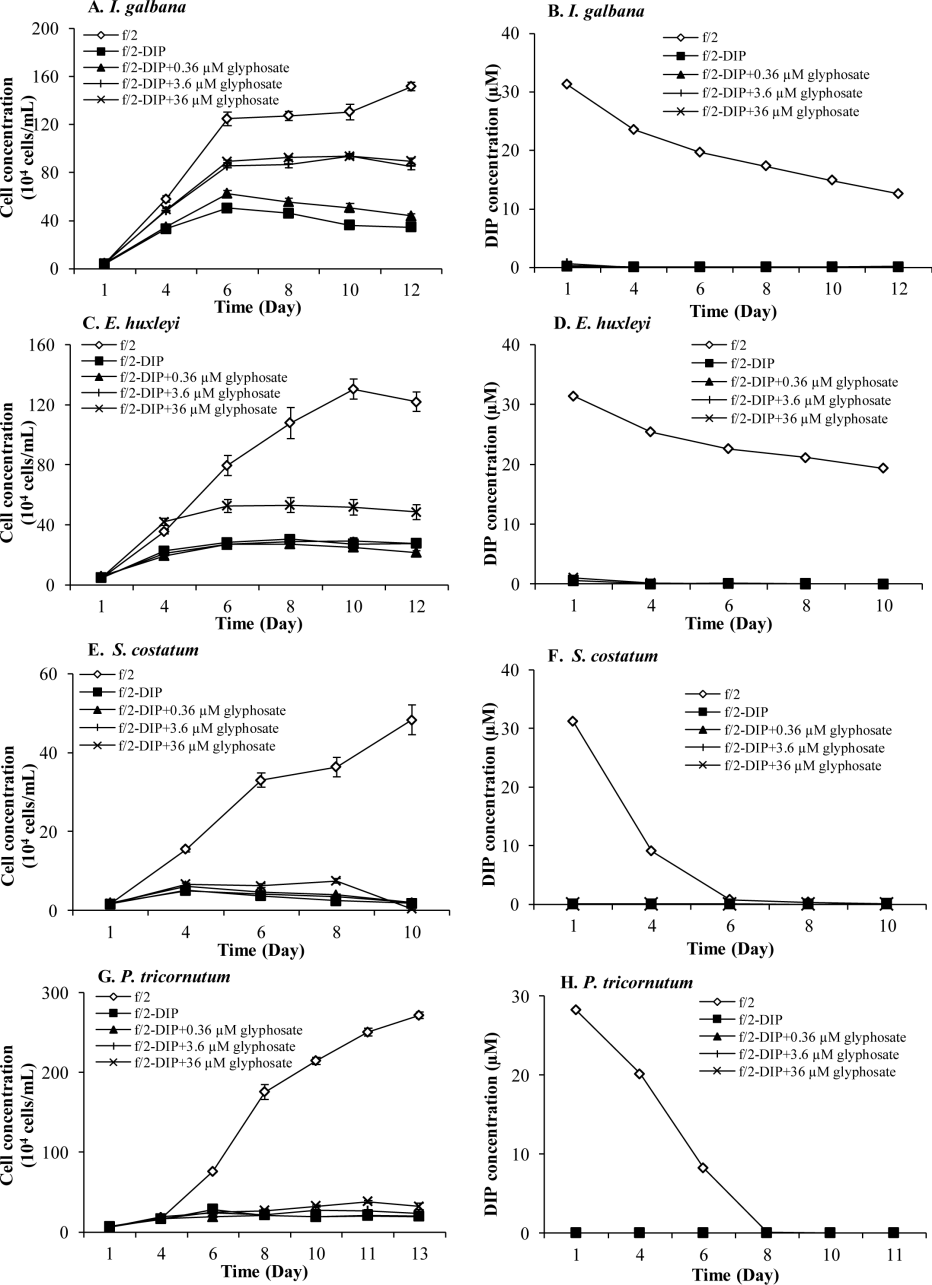
Growth responses of phytoplankton species to different glyphosate concentrations in Group I and Group III. (A) (C) (E) (G) Growth curves Group I (*E*. *huxleyi*, *S*. *costatum*, *P*. *tricornutum*) and Group III (*I*. *galbana*) with different abilities to utilize a wider concentration range of glyphosate (0.36, 3.6 and 36 μM) as sole P-source in the axenic cultures. (B) (D) (F) (H) No DIP release in–DIP+glyphosate cultures compared to–DIP cultures. Error bar represents standard deviation.

We then set up Experiment III (Table 2) trying to verify that in the four glyphosate-promoted species, *I*. *galbana*, *E*. *huxleyi*, *S*. *costatum* and *P*. *tricornutum*, it was the phytoplankton species not the bacteria coexisting in the cultures that were responsible for the glyphosate breakdown to release DIP. Meanwhile, we attempted to explore the responses of the species to varying dosages of glyphosate, particularly to cover possible concentrations in the natural marine ecosystem. We conducted experiments using glyphosate at 0.36, 3.6, and 36 μM in axenic cultures of the four species (Fig 4). For *I*. *galbana*, all the three concentrations of glyphosate supported its growth (Fig 4A) while DIP concentrations in these treatments were almost zero (Fig 4B). The two higher concentrations, 3.6 and 36 μM, lead to almost the same cell yield, which was substantially higher than that in the 0.36-μM glyphosate treatment group, indicating that 3.6-μM glyphosate was sufficient to support glyphosate-based best growth of *I*. *galbana*. However, both the growth rate and cell yield under these two glyphosate conditions were still considerably lower than that in the control group. For the other haptophyte we examined, *E*. *huxleyi*, only 36 μM of glyphosate supported substantially higher growth than the–DIP group, while the lower glyphosate groups were indistinguishable from the–DIP treatment group (Fig 4C). No DIP release to the medium was detected in any of the glyphosate-treated cultures (Fig 4B and 4D). In the case of the two diatom species, *S*. *costatum* and *P*. *tricornutum*, we observed a different dosage response to glyphosate (Fig 4E and 4G) than the non-axenic cultures observed earlier (Fig 1E and 1G). In comparison to the–DIP group, no marked growth enhancement by glyphosate at any of the three concentrations was observed (Fig 4E and 4G). Statistical analysis showed, however, a better growth in the 36-μM glyphosate treatment than under the–DIP condition (*p* < 0.05, RM ANOVA) in both species. All the results in this experiment indicated that these four species described above could utilize glyphosate as sole P-source to support growth but with different efficiencies.

### Toxicity tests of glyphosate

**Fig 5:**
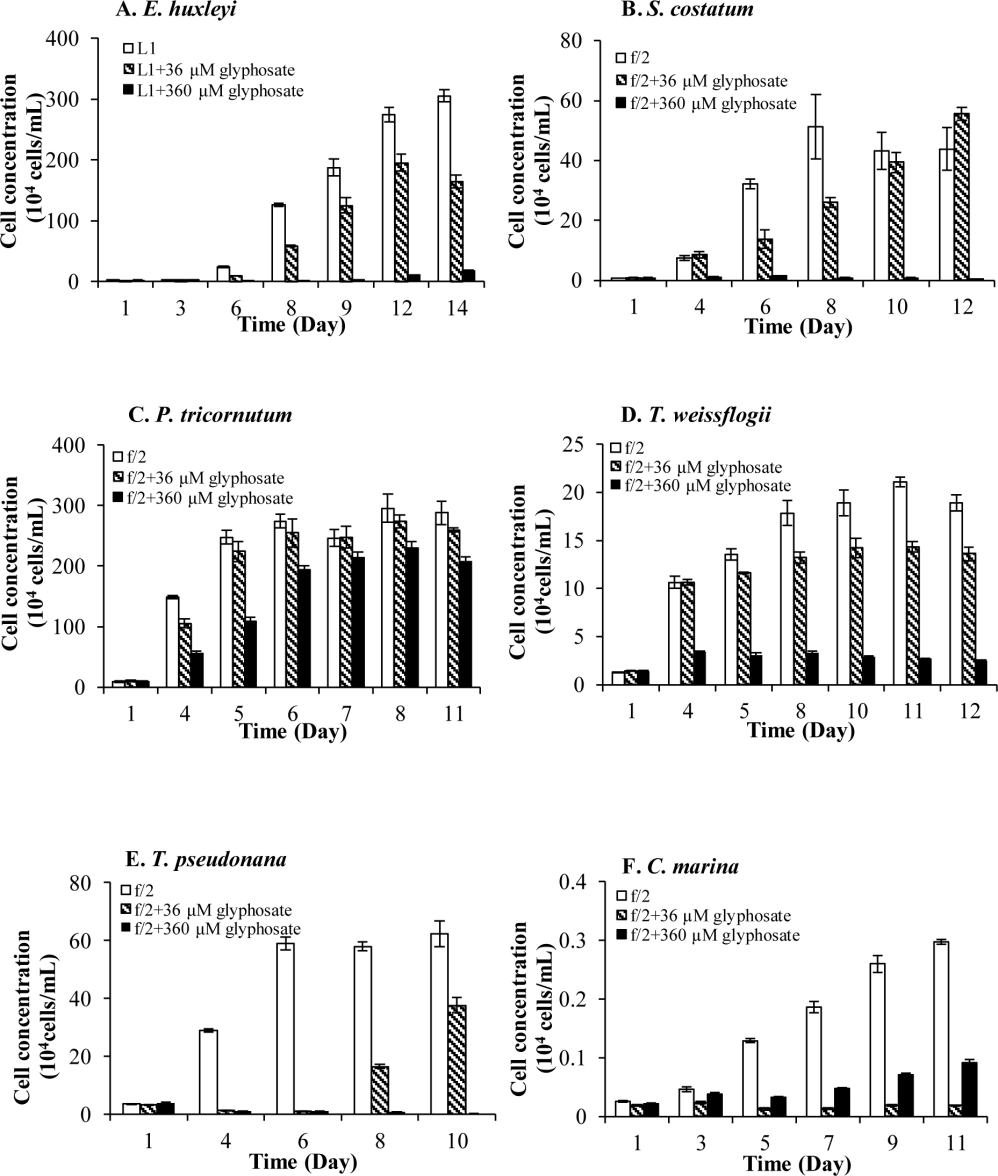
Significantly inhibitory effects of both 36μM and 360μM glyphosate on Group I and Group IV. (A) (B) (C) Growth responses in Group I (*E*. *huxleyi*, *S*. *costatum*, *P*. *tricornutum*) and (D) (E) (F) Group IV (*T*. *weissflogii*, *T*. *pseudonana*, *C*. *marina*) in +DIP cultures. Error bar represents standard deviation.

Toxicity of glyphosate was investigated in Experiment II, using cultures that contained complete nutrients (i.e. same as control) but received addition of glyphosate at 36 and 360 μM concentrations. Fig 5 shows the results of significant growth inhibition under both concentrations of glyphosate relative to the +DIP control group (*p* < 0.05, RM ANOVA) in six of the species examined in this study. While 360-μM glyphosate displayed almost complete growth inhibition in all the species, the toxicity of glyphosate was already remarkable at 36 μM. Comparatively, *T*. *pseudonana* and *C*. *marina* appeared to be particularly sensitive to glyphosate, as extreme growth inhibition was observed even under 36-μM glyphosate (Fig 5E and 5F).

**Fig 6:**
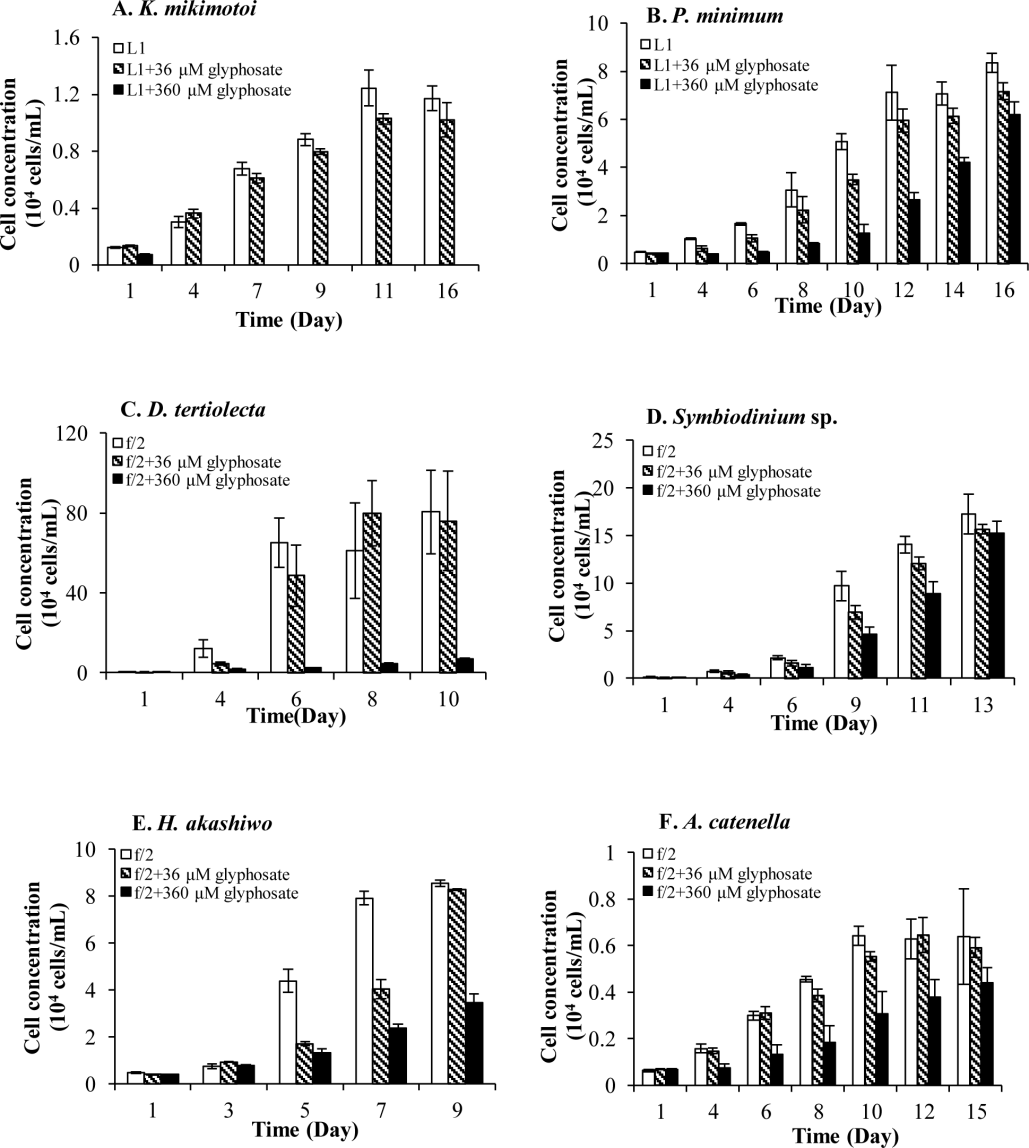
Significantly inhibitory effects of 360μM glyphosate on Group II. (A) (B) (C) (D) (E) (F) showed inhibitory growth responses of different phytoplankton species in Group II (*A*. *catenella*, *P*. *minimum*, *K*. *mikimotoi*, *Symbiodinium* sp., *H*. *akashiwo*, *D*. *tertiolecta*) in +DIP cultures, while 36-μM glyphosate made no difference to their growth. Error bar represents standard deviation.

Another six species displayed more moderate sensitivity to glyphosate because their growth was only inhibited by 360-μM glyphosate (Fig 6). Lethal effects of 360-μM glyphosate were noticeable for *K*. *mikimotoi* and *D*. *tertiolecta* (Fig 6A and 6C) in which no inhibitory effect was observed at 36 μM. In contrast, the other four species steadily grew over time at both glyphosate concentrations although at lower rates than in the +DIP treatment group (Fig 6B, 6D, 6E and 6F), indicating a strong tolerance to glyphosate. The growth rate and biomass yield were higher at the lower glyphosate concentration, exhibiting a dose-dependent partial inhibition.

**Fig 7:**
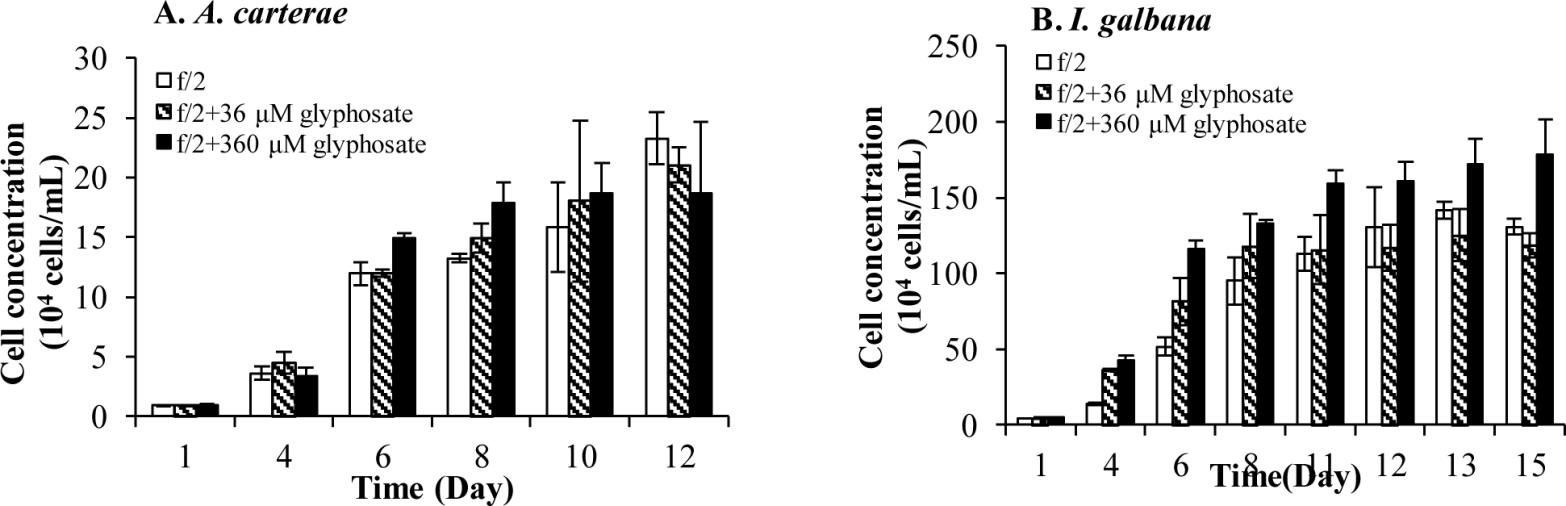
No inhibitory effects of 36 μM and 360μM glyphosate on Group III and Group V. (A) Both 36-μM and 360-μM glyphosate showed no inhibitory effects on Group V (*A*. *carterae*) in the +DIP cultures. (B) Promotion effects of 360-μM glyphosate on Group III (*I*. *galbana*) in +DIP cultures. Error bar represents standard deviation.

The remaining two species, *I*. *galbana* and *A*. *carterae*, exhibited no inhibitory effects of glyphosate. The growth of *A*. *carterae* was not influenced by either 36- or 360-μM glyphosate (Fig 7A) while that of *I*. *galbana* was promoted significantly (*p* < 0.05, RM ANOVA) even under 360-μM glyphosate (Fig 7B). After the initial inoculation, *I*. *galbana* cell density increased to 121%-305% of the control, a result obtained consistently in two separate experiments.

## Discussion

The widely agricultural application of glyphosate as herbicide has led to the presence of this chemical in measurable quantities in lakes and rivers [66,77,78] and it can potentially impact the coastal ocean ecosystem by inhibiting the sensitive species and allowing the tolerant ones to increase. However, how this herbicide may influence marine coastal phytoplankton communities has previously not been documented. In this study we examined the effects of this compound on the growth of fourteen representative phytoplankton species from two contrasting perspectives, as P nutrient (a phosphonate) or growth inhibitor. Our results indicate that responses to glyphosate vary greatly depending on the phytoplankton species.

### Glyphosate as P-source for some species

One of the most remarkable results of our experiments was that four of the fourteen algal species examined in this study, including the haptophytes *I*. *galbana* and *E*. *huxleyi* and the diatoms *S*. *costatum*, and *P*. *tricornutum*, were able to utilize glyphosate as sole P-source to support growth when DIP was depleted. This glyphosate utilizing ability was not due to the mediation of bacteria that may exist in the cultures, because the same results were observed in the axenic cultures. To our knowledge, this is the first report that glyphosate stimulates eukaryotic phytoplankton as sole P-source. However, the growth supported by glyphosate was markedly lower than DIP. The reduced growth rate may be due either to the rate limiting release of phosphate from glyphosate or to partial inhibition of EPSP synthase by glyphosate, which would result in reduction of the biosynthesis of aromatic amino acids [79]. This result was also observed in Rhizobiaceae strains of bacteria, which could utilize glyphosate as sole P-source but only attained small growth; after glyphosate removal population doubling time reduced approximately 50%, indicating the level of glyphosate was efficiently taken up by the cells but was metabolized slowly, resulting in the accumulation of glyphosate inside the cell to an inhibitory level such that removing glyphosate from the medium triggered the cells to grow on the stored glyphosate [72]. These results imply that if the supply rate of glyphosate is sufficient, the four species described above can maintain growth but at lower rates than they would under comparable DIP supplies.

The importance of phosphonates as a P reservoir in the global ocean is by now well-established but information on the molecular strategies employed by marine microorganisms for their utilization remains incomplete [80]. The predominant route for microbial utilization of phosphonates has been thought to be the “C–P lyase” pathway [81] and its presence and activity in marine microorganisms has recently been demonstrated [82]. A complete C-P lyase operon (encoded by *phn* gene family) was recently identified in the cyanobacterium *T*. *erythraeum* [82]. Another cyanobacterium, *Nostoc*, has been shown to be able to transform glyphosate to phosphate for P-source [35]. In the *phn* gene family, *phnD* that encodes the phosphonate binding protein of the ABC-type phosphonate transporter occurs in many of the picocyanobacterial genome sequences [47], indicating the widespread genetic potential in picoplankton to utilize phosphonates. This gene is widely expressed in the Sargasso Sea [48], implying the importance of phosphonate utilization in the oligotrophic ocean. As genomes of *E*. *huxleyi*, *P*. *tricornutum* and *T*. *pseudonana* have been sequenced, we used amino acid sequences encoded by *phn* genes (*phnCDEFGHIJKLM* cluster) in cyanobacteria *Nostoc* sp. PCC7120 as queries to carry out basic local alignment search tool (blast) analysis against these genome databases at Joint Genome Institute (JGI) website to find homologs. Blast analysis at the cutoff e-value of 1.0E-5 revealed that homologs of *phnC*, *phnK* and *phnL*, which encode nucleotide-binding import protein and phosphonate catabolism auxiliary proteins, occur in these species, and each in numerous copies (S1 Table). The genetic potential in *E*. *huxleyi* and *P*. *tricornutum* to utilize glyphosate found in their genomes is consistent with our physiological results. The disagreement in *T*. *pseudonana* between the presence of the genes and the observed inhibitory effects of glyphosate cannot be explained by current data available, but possibly due to its susceptibility to the concentrations of glyphosate employed in this study co-existing with ability to utilize glyphosate at the concentration of 36 μM. Co-existence of glyphosate degrading and shikimate pathway (which is the target of glyphosate inhibition) molecular systems has been documented (see below), but needs to be investigated for this and other species of marine phytoplankton.

### Toxicity of glyphosate in some species

Our results showed that glyphosate could significantly inhibit the growth of the twelve out of the fourteen species we examined at either 36 μM or 360 μM (Table 1, Figs 5 and 6). Even so, these species exhibited different susceptibilities to glyphosate. *E*. *huxleyi*, *S*. *costatum*, *P*. *tricornutum*, *T*. *weissflogii*, *T*. *pseudonana*, and *C*. *marina* showed severe growth inhibition when the herbicide was applied at both 36 μM and 360 μM, indicating relatively high sensitivity to glyphosate (Fig 5). The other six of the species, *K*. *mikimotoi*, *P*. *minimum*, *D*. *tertiolecta*, *Symbiodinium* sp., *H*. *akashiwo*, and *A*. *catenella*, were inhibited by glyphosate only at 360 μM, indicating lower susceptibility or some level of tolerance to glyphosate (Fig 6). Glyphosate at 36 μM was not toxic to these algae probably because no glyphosate was accumulated inside the cells, as in the case of glyphosate-degrading rhizosphere strain, *Enterobacter cloacae* [73]. It has also been reported that glyphosate-resistant horseweed accumulates glyphosate within the vacuole while sensitive plants succumb to the lethal effects of glyphosate largely because glyphosate accumulates in the cytoplasm [83]. Whether these less susceptible algae might use a similar glyphosate sequestration strategy warrants further studies. This finding also indicates that these six less glyphosate-sensitive algae could be exposed to relatively low levels of glyphosate and still maintain a population.

The toxicity of glyphosate mainly comes from its inhibitory effect on EPSP synthase (EC 2.5.1.19), an enzyme involved in the biosynthesis of aromatic amino acids or shikimate pathway [2]. Therefore, the twelve more or less glyphosate-sensitive species may possess the shikimate pathway. We searched for and found EPSP synthase (class I, see below for more information on classification) coding gene *aroA* in the genome database of *E*. *huxleyi* CCMP1516 (NCBI Reference Sequence: XP_005787936.1) in GenBank. It was then used as query to carry out blast analysis against genome database at JGI website to find homologs in other phytoplankton species. As a result, we found *aroA* in the genomes of *P*. *tricornutum* and *T*. *pseudonana* (S1 Table). These results would predict that these three algae are sensitive to glyphosate, which is consistent with our physiological data that both 36 μM and 360 μM glyphosate elicit significant growth inhibition in these species. Of these species, *E*. *huxleyi* possesses both *phn* (phosphonate-utilizing) and EPSP synthase (glyphosate-sensitive) genes.

Due to the lack of relevant genome data, it is unclear what molecular mechanisms confer *A*. *carterae* and *I*. *galbana* the remarkable tolerance to glyphosate observed in this study. The growth of *A*. *carterae* was not affected by glyphosate at either 36 μM or 360 μM and *I*. *galbana* were even promoted at 360 μM. One possibility is that they do not own the shikimate pathway and acquire essential aromatic amino acids from other ways. Another possibility is that they possess a glyphosate-tolerant EPSP synthase. In fact, based on the susceptibility to the herbicide, EPSP synthase has been classified into two groups: class I EPSP synthases from plants and bacteria that are sensitive to the phosphonate, and class II that is of bacterial origin and shows a higher tolerance to glyphosate [84]. Whether the species we examined in this study possess different types of EPSP synthases warrants further investigation.

### Differential responses to glyphosate and ecological implications

Our data show that responses of phytoplankton species to glyphosate vary greatly, which can be classified into five groups (Table 1), ranging from no response (Group V), to monotonically positive (Group III), monotonically negative (Group IV), or variable responses depending on the dosage (Group I and Group II). Group I (*E*. *huxleyi*, *S*. *costatum*, *P*. *tricornutum*) can utilize glyphosate as sole P-source (i.e. in the absence of DIP) but are inhibited by both 36- and 360-μM glyphosate in the presence of DIP. Group II (*K*. *mikimotoi*, *P*. *minimum*, *D*. *tertiolecta*, *Symbiodinium* sp., *H*. *akashiwo* and *A*. *catenella*) are only inhibited by 360-μM glyphosate, but cannot utilize glyphosate as P nutrient at 36 μM concentrations; In Group III (*I*. *galbana*) glyphosate not only can be utilized in the absence of DIP, but also promotes growth in the presence of DIP; that is, *I*. *galbana* can utilize glyphosate whether DIP is present or not. By contrast, Group IV species (*T*. *weissflogii*, *T*. *pseudonana* and *C*. *marina*) were consistently inhibited by glyphosate, while Group V (*A*. *carterae*) exhibited no response to glyphosate.

The above grouping does not follow the phylogenetic relationships of the phytoplankton species, indicating no evolutionary trend in glyphosate utilization or tolerance. As shown in Table 1, except in chlorophyte for which there is only one species included in this study, all other phyla each contains species from more than one glyphosate grouping. Yet it is noteworthy that among all the phyla examined the haptophyte seems to be the most tolerant phylum, considering both *I*. *galbana* and *E*. *huxleyi* possess the ability to use glyphosate as sole P-source in axenic cultures to support growth, and the former exhibited significantly promoted growth even at the higher concentration of 360 μM. However, dinoflagellates and chlorophytes showed a generally moderate tolerance because 36-μM glyphosate neither supported growth as P-source nor inhibited growth as an herbicide, while 360-μM glyphosate inhibited growth eventually. Nevertheless, there was still an exception; *A*. *carterae* showed no inhibition at all and was the most tolerant of all the species examined in this study. In addition, it is clear from Table 1 that most sensitive species, which were inhibited by glyphosate at both 36 and 360 μM, mainly come from the phyla of diatoms and the raphidophyte.

It is intriguing that species in Group I can degrade glyphosate for P source under DIP-deficient condition but sensitive to glyphosate in the presence of DIP. It seems probable that DIP-depleted environment induces the expression of the DOP utilization mechanism so that the cells can obtain P that they need to survive, while the presence of DIP might down-regulate glyphosate degrading enzyme genes resulting in the accumulation of glyphosate and blocking of the shikimate pathway. Although this cannot explain why the cells would still take up glyphosate when there is sufficient DIP, this proposition is however consistent with the fact that *P*. *tricornutum* and *E*. *huxleyi* in Group I have both phosphonate utilization genes and the shikimate pathway enzyme (S1 Table).

The differential responses to glyphosate among different species and different P conditions have potentially significant ecological implications. It is conceivable that depending on the ability to utilize glyphosate and susceptibility to glyphosate toxicity, species dominance in a phytoplankton community can alter with changing environmental conditions, allowing glyphosate-benefiting species (such as Group I and Group III) to thrive (even to form blooms) and sensitive species (such as Group IV) to decline. Consequently, glyphosate discharged into coastal marine environment may potentially impact phytoplankton community structure. This contrasts with the general public perception that glyphosate is environmentally safe, and is consistent with results from several previous studies that demonstrated that glyphosate alone or in combination with the additives used in commercial formulations may be damaging to aquatic biota [17,18,24,85].

Finally, due to their inherent chemical properties (high water solubility but poor solubility in organic solvents and easy to form complexes) and the lack of an accurate method, it is difficult to extract and quantify glyphosate [18,86]. Because of this, glyphosate distribution in the ocean is unclear. This makes it difficult to design experiments with realistic concentrations of glyphosate. As a result, the relatively high concentrations used in this study (0.36 μM to 360 μM) might cause the results to be somewhat different from actual circumstances. Besides, phytoplankton may exhibit different physiological responses due to different conditions of temperature and nutrient and oxygen availability (best growth was observed under 20°C, DIN: DIP = 24:1 for all phytoplankton examined in this study). Nevertheless, the lower end of the concentrations used in our experiments was close to estimated concentrations in the natural environment and that used in previous studies, although we also used unrealistic high concentrations to elicit strong responses. We believe that the pattern of differential responses to glyphosate we observed was robust because some of the experiments were repeated and consistent patterns were obtained. However, this study only demonstrates “potential” rather than real effects of glyphosate on the marine phytoplankton on the ecosystem considering there is no documentation in the literature that shows the prevalent concentration of glyphosate in the aquatic environment, especially in the marine coastal environment. With the physiological responses now documented, the next step would be to analyze the molecular basis of the differential responses of phytoplankton species to glyphosate, including the use of transcriptomic analysis to evaluate the expression level of genes involved in glyphosate metabolism and to effectively assess more subtle impacts of glyphosate on marine coastal ecosystem.

## Conclusions

Based on our results, it is clear that different phytoplankton species respond to the herbicide glyphosate differentially (in a total of five modes), but the differences are not related to taxonomic or phylogenetic affiliations of the species. In some cases, the mode of response is DIP-dependent, as some phytoplankton species are able to utilize glyphosate as sole P-source to support growth only under DIP-depleted conditions, while the toxic effects of glyphosate, exerted by blocking intracellular shikimate pathway, may only occur in DIP-replete cultures. Some species (such as Group I) may experience both the two types of effects while some others (Group V) exhibit no response regardless of DIP conditions.

## Supporting Information

S1 Table Information of phn and EPSP synthase genes detected in the genomes of diatoms and haptophyte. Polypeptides (from phnC to phnL) of *phn* gene cluster are from the cyanobacteria *Nostoc* sp. PCC7120 and responsible for glyphosate transportation and catabolism. And we acquired some copies in *P*. *tricornutum*, *T*. *pseudonana* and *E*. *huxleyi*, which possess the ability to utilize glyphosate as sole P-source in our study. EPSP synthase is the target of glyphosate as an herbicide, and the acquired copies in *P*. *tricornutum* and *T*. *pseudonana* and the sequence in *E*. *huxleyi* are coincided with our results that these species’ growth could be inhibited by glyphosate.
(PDF)

## References

[1] Nandula VK, Reddy KN, Duke SO. Glyphosate-resistant weeds: current status and fiture outlook. Outlooks Pest M. 2005; 16: 183–187.

[2] Herrmann KM, Weaver LM. The shikimate pathway. Annu Rev Plant Biol. 1999; 50: 473–503. 10.1146/annurev.arplant.50.1.473 15012217

[3] Sáenz M, Di Marzio W, Alberdi J, del Carmen Tortorelli M.Effects of technical grade and a commercial formulation of glyphosate on algal population growth. Bull Environ Contamin Toxicol. 1997; 59: 638–644. 10.1007/s001289900527 9307431

[4] Ali A, Fletcher R.Phytotoxic action of glyphosate and amitrole on corn seedlings. Can J Botan. 1978; 56: 2196–2202.

[5] Hernando F, Royuela M, Muñoz-Rueda A, Gonzalez-Murua C. Effect of glyphosate on the greening process and photosynthetic metabolism in Chlorella pyrenoidosa. J Plant Physiol. 1989; 134: 26–31.

[6] Schaffer J, Sebetich M. Effects of aquatic herbicides on primary productivity of phytoplankton in the laboratory. B Environ Contam Tox. 2004; 72: 1032–1037. 10.1007/s00128-004-0347-7 15266702

[7] Annett R, Habibi HR, Hontela A. Impact of glyphosate and glyphosate-based herbicides on the freshwater environment. J App Toxicol. 2014; 34: 458–479. 10.1002/jat.2997 24615870

[8] Duke SO, Powles SB. Glyphosate: a once-in-a-century herbicide. Pest Manag Sci. 2008; 64: 319–325. 10.1002/ps.1518 18273882

[9] Baylis AD. Why glyphosate is a global herbicide: strengths, weaknesses and prospects. Pest Manag Sci. 2000; 56: 299–308.

[10] Giesy JP, Dobson S, Solomon KR. Ecotoxicological risk assessment for Roundup^®^ herbicide In: Giesy JP, Dobson S, Solomon KR, editors. Reviews of Environmental Contamination and Toxicology. Springer New York; 2000 pp. 35–120.

[11] Edge CB, Gahl MK, Thompson DG, Houlahan JE. Laboratory and field exposure of two species of juvenile amphibians to a glyphosate-based herbicide and Batrachochytrium dendrobatidis. Sci Total Environ. 2013; 444: 145–152. 10.1016/j.scitotenv.2012.11.045 23262329

[12] Bowmer KH. Residues of glyphosate in irrigation water. Pestic Sci. 1982; 13: 623–638.

[13] Goldsborough L, Beck A. Rapid dissipation of glyphosate in small forest ponds. Arch Environ Contamin Toxicol. 1989; 18: 537–544.

[14] Feng JC, Thompson DG, Reynolds PE. Fate of glyphosate in a Canadian forest watershed. 1. Aquatic residues and off-target deposit assessment. J Agri Food Chem. 1990; 38: 1110–1118.

[15] Pérez G, Torremorell A, Mugni H, Rodriguez P, Vera MS, Nascimento M, et al Effects of the herbicide Roundup on freshwater microbial communities: a mesocosm study. Ecol Appl. 2007; 17: 2310–2322. 18213971 10.1890/07-0499.1

[16] Peck LS. Organisms and responses to environmental change. Mar Genom. 2011; 4: 237–243. 10.1016/j.margen.2011.07.001 22118635

[17] Vera MS, Lagomarsino L, Sylvester M, Pérez GL, Rodríguez P, Mugni H, et al New evidences of Roundup^®^ (glyphosate formulation) impact on the periphyton community and the water quality of freshwater ecosystems. Ecotoxicology. 2010; 19: 710–721. 10.1007/s10646-009-0446-7 20091117

[18] Annett R, Habibi HR, Hontela A. Impact of glyphosate and glyphosate-based herbicides on the freshwater environment. J Appl Toxicol.2014; 34: 458–479. 10.1002/jat.2997 24615870

[19] Arunakumara K, Walpola BC, Yoon M-H. Metabolism and degradation of glyphosate in aquatic cyanobacteria: A review. African J Microbiol Res. 2013; 7: 4084–4090.

[20] Vendrell E, Ferraz DG, Sabater C, Carrasco JM. Effect of glyphosate on growth of four freshwater species of phytoplankton: a microplate bioassay. Bull Environ Contam Toxicol. 2009; 82: 538–542. 10.1007/s00128-009-9674-z 19266135

[21] Kreutzweiser D, Kingsbury P, Feng J. Drift response of stream invertebrates to aerial applications of glyphosate. Bull Environ Contamin Toxicol. 1989; 42: 331–338. 10.1007/BF01699957 2706342

[22] Folmar LC, Sanders H, Julin A. Toxicity of the herbicide glyphosate and several of its formulations to fish and aquatic invertebrates. Arch Environ Contamin Toxicol. 1979; 8: 269–278. 10.1007/BF01056243 507937

[23] Relyea RA. (2005) The lethal impact of Roundup on aquatic and terrestrial amphibians. Ecol Appl. 2005; 15: 1118–1124.

[24] Uren Webster TM, Laing LV, Florance H, Santos EM. Effects of Glyphosate and its Formulation, Roundup, on Reproduction in Zebrafish (Danio rerio). Environ Sci Technol. 2014; 48: 1271–1279. 10.1021/es404258h 24364672

[25] Servizi J, Gordon R, Martens D. Acute toxicity of Garlon 4 and Roundup herbicides to salmon, Daphnia, and trout. Bull Environ Contamin Toxicol. 1987; 39: 15–22. 10.1007/BF01691783 3607312

[26] Mitchell DG, Chapman PM, Long TJ. Acute toxicity of Roundup^®^ and Rodeo^®^ herbicides to rainbow trout, chinook, and coho salmon. Bull Environ Contamin Toxicol. 1987; 39: 1028–1035. 10.1007/BF01689594 3440140

[27] Stachowski-Haberkorn S, Becker B, Marie D, Haberkorn H, Coroller L, Broise D. Impact of Roundup on the marine microbial community, as shown by an in situ microcosm experiment. Aquat Toxicol. 2008; 89: 232–241. 10.1016/j.aquatox.2008.07.004 18760491

[28] Fiori E, Pistocchi R. Skeletonema marinoi (Bacillariophyceae) sensitivity to herbicides and effects of temperature increase on cellular responses to terbuthylazine exposure. Aquat Toxicol. 2014; 147: 112–120. 10.1016/j.aquatox.2013.12.014 24406202

[29] Ma J, Lin F, Wang S, Xu L. Toxicity of 21 herbicides to the green alga Scenedesmus quadricauda. Bull Environ Contamin Toxicol. 2003; 71: 0594–0601. 10.1007/s00128-003-8521-x 14567587

[30] Van den Brink PJ, Ter Braak CJ. Principal response curves: Analysis of time-dependent multivariate responses of biological community to stress. Environ Toxicol Chem. 1999; 18: 138–148.

[31] Busse MD, Ratcliff AW, Shestak CJ, Powers RF. Glyphosate toxicity and the effects of long-term vegetation control on soil microbial communities. Soil Biol Biochem. 2001; 33: 1777–1789.

[32] Gimsing AL, Borggaard OK, Jacobsen OS, Aamand J, Sørensen J. Chemical and microbiological soil characteristics controlling glyphosate mineralisation in Danish surface soils. Appl Soil Ecol. 2004; 27: 233–242.

[33] Krzysko-Lupicka T, Sudol T. Interactions between glyphosate and autochthonous soil fungi surviving in aqueous solution of glyphosate. Chemosphere. 2008; 71: 1386–1391. 10.1016/j.chemosphere.2007.11.006 18177917

[34] Duke SO. Glyphosate degradation in glyphosate-resistant and -susceptible crops and weeds. J Agric Food Chem. 2011; 59: 5835–5841. 10.1021/jf102704x 20919737

[35] Forlani G, Pavan M, Gramek M, Kafarski P, Lipok J. Biochemical bases for a widespread tolerance of cyanobacteria to the phosphonate herbicide glyphosate. Plant Cell Physiol. 2008; 49: 443–456. 10.1093/pcp/pcn021 18263622

[36] Hove-Jensen B, Zechel DL, Jochimsen B. Utilization of glyphosate as phosphate source: biochemistry and genetics of bacterial carbon-phosphorus lyase. Microbiol Mol Biol Rev. 2014; 78: 176–197. 10.1128/MMBR.00040-13 24600043 PMC3957732

[37] Wu J, Sunda W, Boyle EA, Karl DM. Phosphate depletion in the western North Atlantic Ocean. Science. 2000; 289: 759–762. 10926534 10.1126/science.289.5480.759

[38] Tyrrell T. The relative influences of nitrogen and phosphorus on oceanic primary production. Nature. 1999; 400: 525–531.

[39] Karl DM. Aquatic ecology: Phosphorus, the staff of life. Nature. 2000; 406: 31–33. 10894527 10.1038/35017683

[40] Labry C, Herbland A, Delmas D. The role of phosphorus on planktonic production of the Gironde plume waters in the Bay of Biscay. J Plankton Res. 2000; 24: 97–117.

[41] Schindler D. Evolution of phosphorus limitation in lakes. Science. 1997; 195: 260–262. 10.1126/science.195.4275.260 17787798

[42] Hecky R, Kilham P. Nutrient limitation of phytoplankton in freshwater and marine environments: A review of recent evidence on the effects of enrichment. Limnol Oceanogr. 1988; 33: 796–822.

[43] Orrett K, Karl DM. Dissolved organic phosphorus production in surface seawaters. Limnol Oceanogr. 1987; 32: 383–395.

[44] Suzumura M, Ishikawa K. Characterization of dissolved organic phosphorus in coastal seawater using ultrafiltration and phosphohydrolytic enzymes. Limnol Oceanogr. 1998; 43: 1553–1564.

[45] Clark LL, Ingall ED, Benner R. Marine phosphorus is selectively remineralized. Nature. 1998; 393: 426–426.

[46] Kolowith LC. Composition and cycling of marine organic phosphorus. Limnol Oceanogr. 2001; 46: 309–320.

[47] Ilikchyan IN, McKay RML, Zehr JP, Dyhrman ST, Bullerjahn GS. Detection and expression of the phosphonate transporter gene phnD in marine and freshwater picocyanobacteria. Environ Microbiol. 2009; 11: 1314–1324. 10.1111/j.1462-2920.2009.01869.x 19220397

[48] Ilikchyan IN, McKay RML, Kutovaya OA, Condon R, Bullerjahn GS. Seasonal expression of the picocyanobacterial phosphonate transporter gene phnD in the Sargasso Sea. Front Microbiol. 2010; 1: 135 10.3389/fmicb.2010.00135 21687717 PMC3109553

[49] Sun Y, Wang C. The optimal growth conditions for the biomass production of Isochrysis galbana and the effects that phosphorus, Zn^2+^, CO_2_, and light intensity have on the biochemical composition of Isochrysis galbana and the activity of extracellular CA. Biotechnol Bioproc Engin. 2009; 14: 225–231.

[50] Kamlow M. Molecular Study of Dimethylsulfoniopropionate (DMSP) Metabolism in the Coccolithophore Emiliania huxleyi. M.Sc. Thesis, Norwegian University of Science and Technology. 2013. Available at http://www.diva-portal.org/smash/record.jsf?pid=diva2%3A699830&dswid=-6180

[51] Kooistra WH, Sarno D, Balzano S, Gu H, Andersen RA, Zingone A. Global diversity and biogeography of Skeletonema species (bacillariophyta). Protist. 2008; 159: 177–193. 18042429 10.1016/j.protis.2007.09.004

[52] Sunda WG, Huntsman SA. Iron uptake and growth limitation in oceanic and coastal phytoplankton. Mar Chem. 1995; 50: 189–206.

[53] Sunda WG, Huntsman SA. Interrelated influence of iron, light and cell size on marine phytoplankton growth. Nature. 1997; 390: 389–392.

[54] Armbrust EV, Berges JA, Bowler C, Green BR, Martinez D, Putnam NH, et al The genome of the diatom Thalassiosira pseudonana: ecology, evolution, and metabolism. Science. 2004; 306: 79–86. 15459382 10.1126/science.1101156

[55] Bowler C, Allen AE, Badger JH, Grimwood J, Jabbari K, Kuo A, et al The Phaeodactylum genome reveals the evolutionary history of diatom genomes. Nature. 2008; 456: 239–244. 10.1038/nature07410 18923393

[56] Hallegraeff GM. Ocean climate change, phytoplankton community responses, and harmful algal blooms: a formidable predictive challenge. J Phyco. 2010; 46: 220–235.

[57] Leggat W, Hoegh-Guldberg O, Dove S, Yellowlees D. Analysis of an EST library from the dinoflagellate (Symbiodinium sp.) symbiont of reef-building corals. J Phyco. 2007; 43: 1010–1021.

[58] Berner T, Dubinsky Z, Wyman K, Falkowski PG. Photoadaptation and the Package Effect in Dunaliella-Tertiolecta (Chlorophyceae). J Phyco. 1989; 25: 70–78.

[59] Vassiliev IR, Kolber Z, Wyman KD, Mauzerall D, Shukla VK, Falkowski PG. Effects of Iron Limitation on Photosystem II Composition and Light Utilization in Dunaliella tertiolecta. Plant Physiol. 1995; 109: 963–972. 12228645 10.1104/pp.109.3.963 PMC161398

[60] LeGresley M, McDermott G. Counting chamber methods for quantitative phytoplankton analysis 1st ed. Intergovernmental Oceanographic Commission of ©UNESCO; 2010.

[61] Lin X, Zhang H, Huang B, Lin S. Alkaline Phosphatase Gene Sequence And Transcriptional Regulation By Phosphate Limitation In Amphidinium Carterae (Dinophyceae). J Phyco. 2011; 47: 1110–1120. 10.1111/j.1529-8817.2011.01038.x 27020193

[62] Shi X, Zhang H, Lin S. Tandem repeats, high copy number and remarkable diel expression rhythm of form II RuBisCO in Prorocentrum donghaiense (Dinophyceae). PLoS One. 2013; 8: e71232 10.1371/journal.pone.0071232 23976999 PMC3747160

[63] Lipok J, Studnik H, Gruyaert S. The toxicity of Roundup^®^ 360 SL formulation and its main constituents: glyphosate and isopropylamine towards non-target water photoautotrophs. Ecotox Environ Safe. 2010; 73: 1681–1688. 10.1016/j.ecoenv.2010.08.017 20813408

[64] Peruzzo PJ, Porta AA, Ronco AE. Levels of glyphosate in surface waters, sediments and soils associated with direct sowing soybean cultivation in north pampasic region of Argentina. Environ Pollut. 2008; 156: 61–66. 10.1016/j.envpol.2008.01.015 18308436

[65] Sandrini JZ, Rola RC, Lopes FM, Buffon HF, Freitas MM, Martins C, et al Effects of glyphosate on cholinesterase activity of the mussel Perna perna and the fish Danio rerio and Jenynsia multidentata: In vitro studies. Aquat Toxicol. 2013; 130: 171–173. 10.1016/j.aquatox.2013.01.006 23411353

[66] Saxton MA, Morrow EA, Bourbonniere RA, Wilhelm SW. Glyphosate influence on phytoplankton community structure in Lake Erie. J Great Lakes Res. 2011; 37: 683–690.

[67] Quinn JP, Peden JM, Dick RE. Carbon-phosphorus bond cleavage by Gram-positive and Gram-negative soil bacteria. Appl Microbiol Biot. 1989; 31: 283–287.

[68] Pipke R, Amrhein N. Degradation of the phosphonate herbicide glyphosate by Arthrobacter atrocyaneus ATCC 13752. Appl Environ Microb. 1988; 54: 1293–1296. 10.1128/aem.54.5.1293-1296.1988 PMC202644 16347639

[69] Pipker R, Amerhein N, Jacob GS, Schaefer J, Kishore GM. Metabolism of glyphosate in an Arthrobacter sp. GLP-1. Eur J Biochem. 1987; 165: 267–273. 2439330 10.1111/j.1432-1033.1987.tb11437.x

[70] Talbot HW, Johnson LM, Munnecke DM. Glyphosate utilization byPseudomonas sp. and Alcaligenes sp. isolated from environmental sources. Curr Microbiol. 1984; 10: 255–259.

[71] Moore JK, Braymer HD, Larson AD. Isolation of a Pseudomonas sp. which utilizes the phosphonate herbicide glyphosate. Appl Environ Microb. 1983; 46: 316–320. 10.1128/aem.46.2.316-320.1983 PMC239379 16346357

[72] Liu C-M, McLean P, Sookdeo C, Cannon F. Degradation of the herbicide glyphosate by members of the family Rhizobiaceae. Appl Environ Microb. 1991; 57: 1799–1804. 10.1128/aem.57.6.1799-1804.1991 PMC183471 16348512

[73] Kryuchkova YV, Burygin GL, Gogoleva NE, Gogolev YV, Chernyshova MP, Makarov OE, et al Isolation and characterization of a glyphosate-degrading rhizosphere strain, Enterobacter cloacae K7. Microbiol Res. 2014; 169: 99–105. 10.1016/j.micres.2013.03.002 23545355

[74] Dick R, Quinn J. Glyphosate-degrading isolates from environmental samples: occurrence and pathways of degradation. Appl Microbiol Biot. 1995; 43: 545–550. 10.1007/BF00218464 7632402

[75] Karl DM, Tien G. MAGIC: a sensitive and precise method for measuring dissolved phosphorus in aquatic environments. Limno Oceanogr. 1992; 37: 105–116.

[76] Winer BJ, Brown DR, Michels KM. Statistical principles in experimental design 2nd ed. McGraw-Hill New York; 1971.

[77] Mohr S, Berghahn R, Feibicke M, Meinecke S, Ottenströer T, Schmiedling I, et al Effects of the herbicide metazachlor on macrophytes and ecosystem function in freshwater pond and stream mesocosms. Aquat Toxicol. 2007; 82: 73–84. 17353057 10.1016/j.aquatox.2007.02.001

[78] Mohr S, Feibicke M, Berghahn R, Schmiediche R, Schmidt R. Response of plankton communities in freshwater pond and stream mesocosms to the herbicide metazachlor. Environ Pollut. 2008; 152: 530–542. 17719156 10.1016/j.envpol.2007.07.010

[79] Schulz A, Krüper A, Amrhein N. Differential sensitivity of bacterial 5-enolpyruvylshikimate-3-phosphate synthases to the herbicide glyphosate. FEMS Microbiol Lett. 1985; 28: 297–301.

[80] Villarreal-Chiu JF, Quinn JP, McGrath JW. The genes and enzymes of phosphonate metabolism by bacteria, and their distribution in the marine environment. Front Microbiol. 2012; 3: 19 10.3389/fmicb.2012.00019 22303297 PMC3266647

[81] Ternan NG, Mc Grath JW, Mc Mullan G, Quinn JP. Review: organophosphonates: occurrence, synthesis and biodegradation by microorganisms. World J Microb Biot. 1998; 14: 635–647.

[82] Dyhrman ST, Chappell PD, Haley ST, Moffett JW, Orchard ED, Waterbury JB, et al Phosphonate utilization by the globally important marine diazotroph Trichodesmium. Nature. 2006; 439: 68–71. 16397497 10.1038/nature04203

[83] Ge X, d'Avignon DA, Ackerman JJ, Sammons RD. Rapid vacuolar sequestration: the horseweed glyphosate resistance mechanism. Pest Manag Sci. 2010; 66: 345–348. 10.1002/ps.1911 20063320 PMC3080097

[84] Forlani G, Bertazzini M, Barillaro D, Rippka R.) Divergent properties and phylogeny of cyanobacterial 5-enol-pyruvyl-shikimate-3-phosphate synthases: evidence for horizontal gene transfer in the Nostocales. New Phytol. 2014; 205: 160–171. 10.1111/nph.13022 25229999

[85] Modesto KA, Martinez CB. Effects of Roundup Transorb on fish: hematology, antioxidant defenses and acetylcholinesterase activity. Chemosphere. 2010; 81: 781–787. 10.1016/j.chemosphere.2010.07.005 20684975

[86] Stalikas CD, Konidari CN. Analytical methods to determine phosphonic and amino acid group-containing pesticides. J Chromatogr A. 2001; 907: 1–19. 11217016 10.1016/s0021-9673(00)01009-8

