# Supplementary material for: Differential Growth Responses of Marine Phytoplankton to Herbicide Glyphosate
Source: PLoS One. 2016 Mar 17;11(3):e0151633. doi: 10.1371/journal.pone.0151633 (PMC4795549; doi:10.1371/journal.pone.0151633)
Supplement: S1 Table — Polypeptides (from phnC to phnL) of phn gene cluster are from the cyanobacteria Nostoc sp. PCC7120 and responsible for glyphosate transportation and catabolism. And we acquired some copies in P. tricornutum, T. pseudonana and E. huxleyi, which possess the ability to utilize glyphosate as sole P-source in our study. EPSP synthase is the target of glyphosate as an herbicide, and the acquired copies in P. tricornutum and T. pseudonana and the sequence in E. huxleyi are coincided with our results that these species’ growth could be inhibited by glyphosate. (PDF) [file pone.0151633.s001.pdf]

S1 Table. Information of phn and EPSP synthase genes detected in the genomes of diatoms and haptophyte.

Polypeptide	Function	Query polypeptide accession number	"Hits" in *P. tricornutum*	"Hits" in *T. pseudonana*	"Hits" in *E. huxleyi*
phnC	nucleotide-binding transport protein	NP_486270.1	chr_1:1-100000 + other 18 copies	chr_9:939644-940183 + other 13 copies	scaffold_326:97793-100423 + other 49 copies
phnD	periplasmic binding transport protein	NP_486268.1	-	-	-
phnE	membrane-spanning transport protein	NP_486396.1	-	-	-
		NP_486267.1	-	-	-
phnF	specifying putative regulatory protein	YP_007074233.1	-	-	-
phnI	known enzymatic activity in phosphonate catabolism	NP_486262.1	-	-	-
phnJ		NP_486259.1	-	-	-
phnM		NP_486261.1	-	-	-
		NP_486256.1	-	-	-
phnG	specifying auxiliary polypeptides involved in phosphonate catabolism	NP_486265.1	-	-	-
phnH		NP_486264.1	-	-	-
phnK		NP_486258.1	chr_16:715539-741781 + other 22 copies	chr_10:208100-551469 + other 12 copies	scaffold_819:10647-11183 + other 57 copies
phnL		NP_486257.1	chr_16:715632-741787 + other 13 copies	chr_19a_19:5695-6308 + other 8 copies	scaffold_606:27469-28011 + other 40 copies
EPSP synthase	synthesis of aromatic amino acids	XP 005787936.1	chr_2:915370-916644	chr_3:1993486-1994751	XP 005787936.1
